# Lower mortality risk associated with remdesivir plus corticosteroids vs corticosteroids alone for the treatment of patients hospitalized with SARS-CoV-2 infection in the early and later Omicron periods

**DOI:** 10.1093/ajhp/zxag034

**Published:** 2026-02-09

**Authors:** Alpesh N Amin, Thomas Oppelt, Aastha Chandak, Robert L Gottlieb, Chidinma Chima-Melton, Natasha N Pettit, Andre C Kalil

**Affiliations:** Department of Medicine, School of Medicine, University of California Irvine, Irvine, CA, USA; Medical Affairs, Gilead Sciences, Foster City, CA, USA; Evidence and Access, Certara, Radnor, PA, USA; Department of Internal Medicine, Baylor University Medical Center, Baylor Scott & White Health, Dallas, TX, USA; Burnett School of Medicine at TCU, Fort Worth, TX, USA; Cardiopulmonary Division, Pipeline Health, Los Angeles, CA; Tele-ICU Inc., Los Angeles, CA, USA; Department of Pharmacy, UChicago Medicine, Chicago, IL, USA; Division of Infectious Diseases, Department of Internal Medicine, University of Nebraska Medical Center, Omaha, NE, USA

**Keywords:** corticosteroids, mortality, real-world evidence, remdesivir, SARS-CoV-2

## Abstract

**Purpose:**

Severe acute respiratory syndrome coronavirus 2 (SARS-CoV-2) continues to pose a risk to vulnerable populations. This retrospective study compared the effectiveness of remdesivir plus corticosteroids (CCS) versus CCS alone in patients hospitalized with COVID-19 from December 2021 to December 2024.

**Methods:**

Data were extracted from a large, geographically-diverse US Premier Healthcare Database for adults hospitalized for COVID-19. Exclusion criteria included pregnancy, incomplete data, transfer from another hospital or hospice care, death/discharge during the baseline period, elective procedure admissions, patients without supplemental oxygen in hospitals that did not report charges for low-flow oxygen, and patients on extracorporeal membrane oxygenation. Propensity score matching was used to balance the distribution of underlying confounders in the two treatment groups. A Cox proportional hazards model was used to assess time to 14- and 28-day inpatient all-cause mortality.

**Results:**

A total of 104,900 patients were initiated on remdesivir plus CCS and 66,016 were initiated on CCS alone in the first 2 days of hospitalization. Unadjusted 14- and 28-day mortality rates were lower for remdesivir-treated patients versus patients who did not receive remdesivir during hospitalization. Remdesivir plus CCS initiation upon admission for COVID-19 (in the total population) was associated with a significantly lower mortality rate (*P* < 0.0001) (in the overall Omicron period) at both 14 and 28 days, with an adjusted hazard ratio (95% confidence interval) of 0.77 (0.74-0.80) and 0.79 (0.77-0.82), respectively, versus CCS alone. Results were similar for the total population in the early and later Omicron periods.

**Conclusion:**

Remdesivir plus CCS was associated with a significant reduction in inpatient all-cause mortality relative to CCS alone in patients hospitalized for COVID-19 across 3 years of the Omicron period, illustrating the utility of the most recent real-world evidence to help inform treatment recommendations for inpatient providers treating patients with SARS-CoV-2 infection.

Key Points

- Administration of remdesivir plus corticosteroids versus corticosteroids alone significantly decreased all-cause in-hospital mortality at 14 and 28 days in patients hospitalized for SARS-CoV-2 infection from December 2021 to December 2024 with or without supplemental oxygen requirements.
- These results further expand on the findings from previous studies demonstrating the effectiveness of remdesivir in vulnerable patient populations hospitalized for COVID-19.
- This study also illustrates the continued utility of real-world evidence to help inform treatment recommendations for inpatient providers treating patients with SARS-CoV-2 infection.

Severe acute respiratory syndrome coronavirus 2 (SARS-CoV-2) continues to pose a risk to vulnerable patient populations with immunocompromising conditions^2^ or chronic diseases^3^ and patients who are 65 years of age or older.^4^ In addition, these populations often take several medications, which can make avoidance and management of drug-drug interactions in patients hospitalized for SARS-CoV-2 infection critically important.^5^ Since the end of the coronavirus disease 2019 (COVID-19) pandemic era, the first line of care for SARS-CoV-2–infected patients has shifted from infectious disease specialists to inpatient providers, including clinical pharmacists. In the current era, effective treatment recommendations for populations at risk for severe SARS-CoV-2 infection are being assessed using real-world evidence.^6^

The efficacy and effectiveness of the antiviral remdesivir in the treatment of patients hospitalized for SARS-CoV-2 infection has been demonstrated using randomized controlled trials^7–12^ and real-world evidence, respectively. In real-world analyses that included data through the early Omicron subvariant period, administration of remdesivir continued to be associated with decreased mortality in patients who required any supplemental oxygen^13^ and in those who did not require supplemental oxygen.^14^ Remdesivir also reduced the likelihood of hospital readmission.^15^ In the RECOVERY trial, dexamethasone showed a mortality benefit in patients hospitalized for SARS-CoV-2 infection receiving supplemental oxygen or invasive mechanical ventilation.^16^ However, there was increased mortality when patients not receiving supplemental oxygen received dexamethasone.^16^ In a recent study, remdesivir plus dexamethasone was associated with a significant reduction in 14- and 28-day mortality when compared to dexamethasone alone in patients hospitalized for SARS-CoV-2 infection across all levels of baseline respiratory support.^17^ Despite this real-world evidence, as well as guideline recommendations, many patients hospitalized for SARS-CoV-2 infection continue to receive dexamethasone alone without the addition of an antiviral.^17^

This study provides updated real-world evidence to continue to inform and support inpatient providers who are making clinical decisions for appropriate treatment of vulnerable patients who continue to be hospitalized for SARS-CoV-2 infection. In this analysis, we examined the effect of the antiviral remdesivir on mortality rates in patients hospitalized for SARS-CoV-2 infection in the overall Omicron period (December 2021-December 2024) and—for the first time, to our knowledge—compared the mortality rates in the early (December 2021-December 2022) and more recent, later (January 2023-December 2024) Omicron periods. We also extend the findings from previous studies comparing remdesivir plus dexamethasone versus dexamethasone alone to include patients receiving remdesivir plus corticosteroids (CCS) versus CCS alone.^17^ Therefore, through this analysis we aim to provide updated real-world evidence to support clinical decision-making for vulnerable populations with SARS-CoV-2 infection.

## Methods

### Study design and data source

We conducted a retrospective study to compare the effectiveness of remdesivir plus CCS versus CCS alone in patients hospitalized for COVID-19 during the overall Omicron period (December 2021-December 2024) and to stratify results into the early (December 2021-December 2022) and the more recent, later (January 2023-December 2024) Omicron periods. The data source for this study was the Premier Healthcare Database, a large, geographically diverse, Health Insurance Portability and Accountability Act–compliant, all-payor hospital administrative billing database including approximately 25% of US hospitalizations every year.^18^ The database captures patient demographics, hospital characteristics, admission and discharge diagnoses, and billing information for services.

### Study population

Adult patients hospitalized with a primary discharge diagnosis of COVID-19 (International Classification of Diseases, 10th Revision, Clinical Modification code U07.1)^1^ flagged as “present-on-admission” from December 2021 to December 2024, during the Omicron period, were included in the study. The study population was subdivided into 3 periods: the overall, early (December 2021-December 2022), and later (January 2023-December 2024) Omicron periods. Treatment groups included those who were initiated on remdesivir plus CCS or CCS alone in the first 2 days of hospitalization (designated as the baseline period). Patients in each treatment group were further characterized according to the absence or presence of supplemental oxygen charges, as previous studies have shown patients not requiring supplemental oxygen to have higher mortality rates when given CCS alone.^16^ Patients with no supplemental oxygen charges (NSOc) were included only from hospitals affirmatively demonstrated to charge for supplemental oxygen. Patients requiring any level of supplemental oxygen were identified by the corresponding charges. Data on prior treatment is not available in this inpatient billing database since the patient record begins on the first day of the hospitalization; as such, prior COVID-19 treatment in the study population could not be assessed and patients are included regardless of prior treatments. Exclusion criteria included pregnancy, incomplete data, transfer from another hospital or hospice care, death/discharge during the baseline period, admission for elective procedures, patients in hospitals that did not report charges for low-flow oxygen, and patients on extracorporeal membrane oxygenation upon admission.

### Study outcomes and covariates

Baseline patient characteristics and covariates are shown in Table 1 and include demographic information, primary payor, hospital characteristics such as ward on admission, other treatments at baseline, and baseline supplemental oxygen, including NSOc or any supplemental oxygen (ie, low-flow oxygen, high-flow oxygen/noninvasive ventilation, and invasive mechanical ventilation). Supplemental oxygenation status was determined as the highest level of support recorded in the billing records within the first 2 days of the hospitalization, ie, the same time period during which treatment with remdesivir was identified. Patients were assessed from the day after the baseline period until day 28 or until discharge as expired or to hospice care, transfer to another hospital, or addition of remdesivir after the baseline period in the CCS-alone group (as a per-protocol approach of treatment group assignment), whichever came first. All-cause inpatient mortality was assessed at 14 and 28 days post baseline. This approach has been previously published.^17^

**Table 1.** Baseline Demographics and Hospital Characteristics of Patients Hospitalized for COVID-19 From December 2021 to December 2024

Characteristic	Before PS matching			After PS matching		
	CCS alone N = 66,016	Remdesivir + CCS N = 104,900	Absolute SMD	CCS alone N = 57,765	Remdesivir + CCS N = 57,765	Absolute SMD
**Age group, years**						
18-49	5,314 (8.0)	9,396 (9.0)	0.04	4,142 (7.2)	4,142 (7.2)	0.00
50-64	13,799 (20.9)	22,337 (21.3)		11,618 (20.1)	11,618 (20.1)	
≥65	46,903 (71.0)	73,167 (69.7)		42,005 (72.7)	42,005 (72.7)	
**Female sex**	33,978 (51.5)	53,916 (51.4)	0.00	29,624 (51.3)	29,782 (51.6)	0.01
**Race**						
White	51,210 (77.6)	81,471 (77.7)	0.09	45,214 (78.3)	45,466 (78.7)	0.04
Black	9,338 (14.1)	13,025 (12.4)		7,685 (13.3)	7,568 (13.1)	
Asian	1,011 (1.5)	2,331 (2.2)		919 (1.6)	880 (1.5)	
Other	4,457 (6.8)	8,073 (7.7)		3,947 (6.8)	3,851 (6.7)	
**Ethnicity**						
Hispanic	5,014 (7.6)	10,713 (10.2)	0.07	4,441 (7.7)	4,377 (7.6)	0.04
Non-Hispanic	56,213 (85.2)	87,394 (83.3)		49,340 (85.4)	49,569 (85.8)	
Unknown	4,789 (7.3)	6,793 (6.5)		3,984 (6.9)	3,819 (6.6)	
**Primary payor**						
Commercial	9,031 (13.7)	16,169 (15.4)	0.05	7,960 (13.8)	7,901 (13.7)	0.00
Medicare	48,142 (72.9)	74,489 (71.0)		42,489 (73.6)	42,557 (73.7)	
Medicaid	5,285 (8.0)	9,006 (8.6)		4,311 (7.5)	4,299 (7.4)	
Other	3,558 (5.4)	5,236 (5.0)		3,005 (5.2)	3,008 (5.2)	
**Admission source**						
Transfer from SNF or ICF	2,294 (3.5)	4,023 (3.8)	0.02	2,032 (3.5)	2,001 (3.5)	0.00
**Hospital size, No. of beds**						
<100	5,407 (8.2)	8,175 (7.8)	0.09	4,854 (8.4)	4,757 (8.2)	0.06
100-199	11,475 (17.4)	18,503 (17.6)		10,081 (17.5)	10,178 (17.6)	
200-299	13,635 (20.7)	21,058 (20.1)		12,025 (20.8)	11,854 (20.5)	
300-399	12,923 (19.6)	18,522 (17.7)		11,171 (19.3)	10,917 (18.9)	
400-499	7,275 (11.0)	10,395 (9.9)		5,994 (10.4)	6,419 (11.1)	
≥500	15,301 (23.2)	28,247 (26.9)		13,640 (23.6)	13,640 (23.6)	
**Hospital location**						
Urban	56,306 (85.3)	91,632 (87.4)	0.06	49,462 (85.6)	49,489 (85.7)	0.00
Rural	9,710 (14.7)	13,268 (12.6)		8,303 (14.4)	8,276 (14.3)	
**Teaching hospital**	28,042 (42.5)	47,077 (44.9)	0.05	24,396 (42.2)	24,500 (42.4)	0.00
**Region**						
Midwest	16,710 (25.3)	24,253 (23.1)	0.18	14,565 (25.2)	14,375 (24.9)	0.03
Northeast	6,981 (10.6)	17,822 (17.0)		6,433 (11.1)	6,483 (11.2)	
South	35,041 (53.1)	50,004 (47.7)		30,198 (52.3)	30,384 (52.6)	
West	7,284 (11.0)	12,821 (12.2)		6,569 (11.4)	6,523 (11.3)	
**Comorbid conditions**						
Obesity	20,314 (30.8)	32,244 (30.7)	0.00	17,518 (30.3)	17,556 (30.4)	0.00
Chronic pulmonary disease	27,792 (42.1)	44,219 (42.2)	0.00	24,076 (41.7)	24,291 (42.1)	0.01
Cardiovascular disease	58,799 (89.1)	91,282 (87.0)	0.06	51,289 (88.8)	51,219 (88.7)	0.00
Diabetes	26,877 (40.7)	40,738 (38.8)	0.04	23,157 (40.1)	23,085 (40.0)	0.00
Renal disease	22,701 (34.4)	26,739 (25.5)	0.20	18,418 (31.9)	18,260 (31.6)	0.01
Cancer	4,655 (7.1)	7,836 (7.5)	0.02	4,149 (7.2)	4,111 (7.1)	0.00
**Immunocompromising condition**	12,122 (18.4)	19,016 (18.1)	0.01	10,404 (18.0)	10,359 (17.9)	0.00
**Hospital ward upon admission**						
General ward	54,307 (82.3)	85,177 (81.2)	0.03	48,023 (83.1)	48,367 (83.7)	0.02
ICU/step-down unit	11,709 (17.7)	19,723 (18.8)		9,742 (16.9)	9,398 (16.3)	
**Admission diagnosis**						
Sepsis	336 (0.5)	406 (0.4)	0.02	255 (0.4)	263 (0.5)	0.00
Pneumonia	5,586 (8.5)	7,603 (7.2)	0.05	4,429 (7.7)	4,454 (7.7)	0.00
**Other treatments in the first 2 days of hospitalization**						
Anticoagulants	50,223 (76.1)	84,662 (80.7)	0.11	45,078 (78.0)	45,201 (78.2)	0.01
Convalescent plasma	34 (0.1)	110 (0.1)	0.00	32 (0.1)	29 (0.1)	0.00
Corticosteroids other than dexamethasone	19,576 (29.7)	25,858 (24.7)	0.02	15,724 (27.2)	15,866 (27.5)	0.00
Baricitinib	4,863 (7.4)	5,882 (5.6)	0.03	3,924 (6.8)	3,946 (6.8)	0.00
Tocilizumab	2,093 (3.2)	3,819 (3.6)	0.07	1,873 (3.2)	1,847 (3.2)	0.00
Oral antivirals	996 (1.5)	250 (0.2)	0.14	85 (0.1)	84 (0.1)	0.00
**Supplemental oxygen requirements in the first 2 days of hospitalization^a^**						
NSOc	26,048 (39.5)	39,669 (37.8)	0.10	22,949 (39.7)	22,949 (39.7)	0.00
LFO	24,024 (36.4)	38,576 (36.8)		21,512 (37.2)	21,512 (37.2)	
HFO/NIV	13,050 (19.8)	23,713 (22.6)		11,699 (20.3)	11,699 (20.3)	
IMV	2,894 (4.4)	2,942 (2.8)		1,605 (2.8)	1,605 (2.8)	
**Omicron period**						
Early (Dec 2021-Dec 2022)	45,383 (68.7)	68,634 (65.4)	0.07	40,712 (70.5)	40,712 (70.5)	0.00
Later (Jan 2023-Dec 2024)	20,633 (31.3)	36,266 (34.6)		17,053 (29.5)	17,053 (29.5)	

Abbreviations: CCS, corticosteroids; COVID-19, coronavirus disease 2019; HFO/NIV, high-flow oxygen/noninvasive ventilation; ICF, intermediate care facility; ICU, intensive care unit; IMV, invasive mechanical ventilation; LFO, low-flow oxygen; NSOc, no supplemental oxygen charges; PS, propensity score; SMD, standardized mean difference; SNF, skilled nursing facility.

Data is presented as No. (%), unless otherwise indicated.

^a^LFO, HFO/NIV, IMV collectively referred to as any supplemental oxygen.

### Statistical analysis

Analyses were performed for the total study population and by baseline supplemental oxygen requirements in 2 groups: NSOc and any supplemental oxygen. Propensity scores (PSs) were estimated using separate logistic regression models for NSOc and any supplemental oxygen and included baseline covariates (Table 1). For the primary analysis, the derived PSs were used to balance the distribution of underlying confounders in the treatment groups. Details of the PS methodology used in this study have been published previously.^17^ Briefly, a 1:1 preferential within-hospital matching approach without replacement, with a caliper distance of 0.2 times the standard deviation of the logit of the PS, was used, including baseline covariates. Key study variables are defined in supplementary eTable 1. A sensitivity analysis was performed using stabilized inverse probability of treatment weighting (IPTW) to PS matching.^19^ In the IPTW approach, the extreme PS values (<0.05 and >0.95) were trimmed. Balance between the two treatment groups was assessed at a threshold of absolute standardized mean difference (SMD) of <0.15.

A Cox proportional hazards model was used to assess time to all-cause inpatient mortality at 14 and 28 days. Adjusted hazard ratios (aHRs) and 95% confidence intervals (CIs) were derived. The models were adjusted for hospital-level cluster effects, as previously published.^17^

This study was reviewed by Advarra Institutional Review Board (protocol #Pro00091215) and was determined to be exempt from oversight in accordance with the Department of Health and Human Services regulations found at 45 CFR 46.104(d)(4).

## Results

### Study population demographics

**Figure 1.**
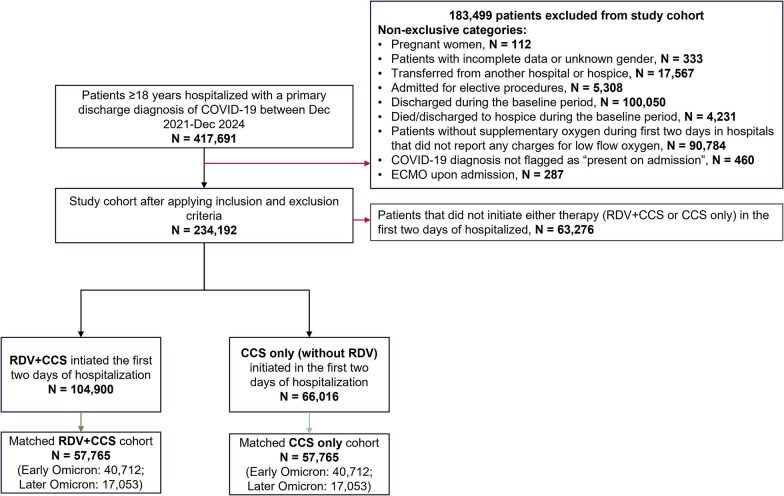
Study flow diagram. CCS indicates corticosteroids; COVID-19, coronavirus disease 2019; ECMO, extracorporeal membrane oxygenation; RDV, remdesivir.

The study included 234,192 patients hospitalized for COVID-19 during the Omicron period from December 2021 to December 2024 (Figure 1). A total of 104,900 patients were initiated on remdesivir plus CCS and 66,016 on CCS alone in the first 2 days of hospitalization. After 1:1 PS matching, 57,765 patients who received remdesivir plus CCS were matched to 57,765 patients who received CCS alone. After matching, 40,712 patients were from the early Omicron period and 17,053 were from the later Omicron period in each treatment group. Specifically, of the 57,765 patients in the CCS group, 5,260 (9.1%) were initiated on remdesivir after the first 2 days of hospitalization and were censored at the time of remdesivir initiation.

Baseline demographics and hospital characteristics for the study population before and after PS matching are shown in Table 1. Before matching, most patients in the remdesivir plus CCS and CCS-alone cohorts were 65 years of age or older (69.7% and 71.0%, respectively), female (51.4% and 51.5%), White (77.7% and 77.6%), and non-Hispanic (83.3% and 85.2%). After matching, patient characteristics were well balanced between groups. Most patients (60.3%) received supplemental oxygen in the first 2 days of hospitalization versus no supplemental oxygen. In the overall cohort before PS matching, the median duration of CCS treatment was 6 days (interquartile range [IQR], 4-9 days) for patients receiving CCS only and 6 days (4-8 days) for patients receiving remdesivir plus CCS, while the median (IQR) duration of remdesivir treatment was 5 days (3-5 days) for patients receiving remdesivir plus CCS. After PS matching, the median (IQR) duration of CCS treatment was 6 days (4-9 days) for both groups, while the median duration of remdesivir treatment was 5 days (3-5 days) for patients receiving remdesivir plus CCS. Data on duration of treatment for subgroups of patients based on supplemental oxygen use are shown in Table 2.

**Table 2.** Treatment Duration of CCS Alone or Remdesivir Plus CCS Before and After PS Matching

	Before PS matching		After PS matching	
	CCS alone	Remdesivir + CCS	CCS alone	Remdesivir + CCS
**Overall cohort**	**(N = 66,016)**	**(N = 104,900)**	**(N = 57,765)**	**(N = 57,765)**
Remdesivir duration, median (IQR), days	NA	5 (3-5)	NA	5 (3-5)
Corticosteroid duration, median (IQR), days	6 (4-9)	6 (4-8)	6 (4-9)	6 (4-9)
**NSOc subgroup**	**(n = 26,048)**	**(n = 39,669)**	**(n = 22,949)**	**(n = 22,949)**
Remdesivir duration, median (IQR), days	NA	5 (3-5)	NA	5 (3-5)
Corticosteroid duration, median (IQR), days	5 (3-7)	5 (4-7)	5 (3-7)	5 (4-7)
**Any supplemental oxygen subgroup**	**(n = 39,968)**	**(n = 65,231)**	**(n = 34,816)**	**(n = 34,816)**
Remdesivir duration, median (IQR), days	NA	5 (4-5)	NA	5 (4-5)
Corticosteroid duration, median (IQR), days	6 (4-10)	6 (5-9)	6 (4-10)	6 (5-10)

Abbreviations: CCS, corticosteroid; IQR, interquartile range; NA, not applicable; NSOc, no supplemental oxygen charges.

### Total study population

**Figure 2.**
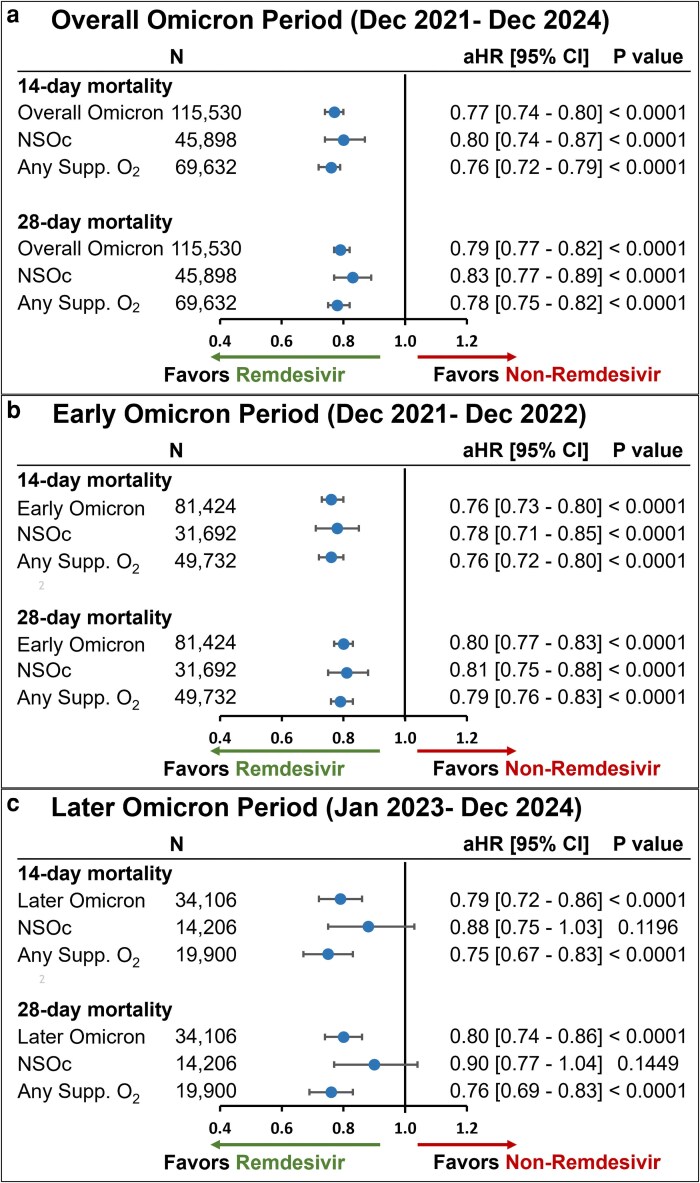
Forest plots for 14- and 28-day mortality in patients hospitalized for coronavirus disease 2019 (COVID-19) receiving remdesivir plus corticosteroids (CCS) versus CCS alone in the overall (a), early (b), and later (c) Omicron periods. Estimates were adjusted for admission month and time-varying treatment with other COVID-19 medications (baricitinib, tocilizumab, and oral antivirals). aHR indicates adjusted hazard ratio; CI, confidence interval; NSOc, no supplemental oxygen charges; Supp., supplemental.

The unadjusted all-cause inpatient mortality rate in the crude population prior to PS matching was consistently lower in the remdesivir group versus the non-remdesivir group in the overall Omicron group as well as the NSOc and any supplemental oxygen subgroups (supplementary eTable 2). Post PS matching, this mortality benefit in the remdesivir group remained consistent. For patients hospitalized for COVID-19 in the overall Omicron period, in the PS-matched cohort (N = 57,765), the unadjusted mortality rate was lower for those administered remdesivir versus those who did not receive remdesivir during hospitalization (7.6% versus 8.8%, respectively, at 14 days and 10.1% versus 11.3%, respectively at 28 days) (supplementary eTable 3). Remdesivir plus CCS was associated with a significantly lower mortality risk versus CCS alone (*P* < 0.0001) in the overall Omicron period, with an aHR (95% CI) at 14 and 28 days of 0.77 (0.74-0.80) and 0.79 (0.77-0.82), respectively (Figure 2a).

For patients hospitalized for COVID-19 in the early Omicron period, in the PS-matched cohort (N = 40,712), the unadjusted mortality rate was lower for those administered remdesivir in the first 2 days of hospitalization versus those who did not receive remdesivir during hospitalization (8.4% versus 9.8%, respectively at 14 days and 11.6% versus 12.8%, respectively at 28 days) (supplementary eTable 3). Remdesivir plus CCS was associated with a significantly lower mortality risk versus CCS alone (*P* < 0.0001) in the early Omicron period, with an aHR (95% CI) at 14 and 28 days of 0.76 (0.73-0.80) and 0.80 (0.77-0.83), respectively (Figure 2b).

For patients hospitalized for COVID-19 in the later Omicron period, in the PS-matched cohort (N = 17,053), the unadjusted mortality rate was lower for those administered remdesivir in the first 2 days of hospitalization versus those who did not receive remdesivir during hospitalization (5.6% versus 6.5%, respectively, at 14 days and 6.7% versus 7.7%, respectively, at 28 days) (supplementary eTable 3). Remdesivir plus CCS was associated with a significantly lower mortality risk versus CCS alone (*P* < 0.0001) in the later Omicron period, with an aHR (95% CI) at 14 and 28 days of 0.79 (0.72-0.86) and 0.80 (0.74-0.86), respectively (Figure 2c).

Similar results were obtained with the IPTW sensitivity analysis across all Omicron periods in the total study population (supplementary eTable 4).

### Study population with NSOc

For patients hospitalized for COVID-19 in the overall Omicron period, in the PS-matched cohort with NSOc (N = 22,949), the unadjusted mortality rate was lower for those administered remdesivir in the first 2 days of hospitalization versus those who did not receive remdesivir during hospitalization (5.0% versus 5.5%, respectively at 14 days and 6.4% versus 6.8%, respectively at 28 days) (supplementary eTable 3). Remdesivir plus CCS was associated with a significantly lower mortality risk versus CCS alone (*P* < 0.0001) in the overall Omicron period, with an aHR (95% CI) at 14 and 28 days of 0.80 (0.74-0.87) and 0.83 (0.77-0.89), respectively, in patients with NSOc (Figure 2a).

For patients hospitalized for COVID-19 in the early Omicron period, in the PS-matched cohort with NSOc (N = 15,846), the unadjusted mortality rate was lower for those administered remdesivir in the first 2 days of hospitalization versus those who did not receive remdesivir during hospitalization (5.4% versus 6.0%, respectively, at 14 days and 7.1% versus 7.6%, respectively, at 28 days) (supplementary eTable 3). Remdesivir plus CCS was associated with a significantly lower mortality risk versus CCS alone (*P* < 0.0001) in the early Omicron period, with an aHR (95% CI) at 14 and 28 days of 0.78 (0.71-0.85) and 0.81 (0.75-0.88), respectively, in patients with NSOc (Figure 2b).

For patients hospitalized for COVID-19 in the later Omicron period, in the PS-matched cohort with NSOc (N = 7,103), the unadjusted mortality rate was lower for those administered remdesivir in the first 2 days of hospitalization versus those who did not receive remdesivir during hospitalization (4.1% versus 4.3%, respectively at 14 days and 4.8% versus 5.0%, respectively at 28 days) (supplementary eTable 3). Remdesivir plus CCS was associated with a lower point-estimated mortality risk versus CCS alone in the later Omicron period, with an aHR (95% CI) at 14 and 28 days of 0.88 (0.75-1.03) (*P* = 0.12) and 0.90 (0.77-1.04) (*P* = 0.14), respectively, in patients with NSOc (Figure 2c).

Results obtained with the IPTW sensitivity analysis of mortality risk for remdesivir plus CCS versus CCS alone in the population with NSOc in the later Omicron period were statistically significant (14-day mortality, *P* = 0.0075; and 28-day mortality, *P* = 0.0071) (supplementary eTable 5).

### Study population with any supplemental oxygen requirements

For patients hospitalized for COVID-19 in the overall Omicron period, in the PS-matched cohort with any supplemental oxygen (N = 34,816), the unadjusted mortality rate was lower for those administered remdesivir in the first 2 days of hospitalization versus those who did not receive remdesivir during hospitalization (9.3% versus 11.0%, respectively at 14 days and 12.6% versus 14.2%, respectively at 28 days) (supplementary eTable 3). Remdesivir plus CCS was associated with a significantly lower mortality risk versus CCS alone (*P* < 0.0001) in the overall Omicron period, with an aHR (95% CI) at 14 and 28 days of 0.76 (0.72-0.79) and 0.78 (0.75-0.82), respectively, in patients with any supplemental oxygen (Figure 2a).

For patients hospitalized for COVID-19 in the early Omicron period in the PS-matched cohort with any supplemental oxygen (N = 24,866), the unadjusted mortality rate was lower for those administered remdesivir in the first 2 days of hospitalization versus those who did not receive remdesivir during hospitalization (10.4% versus 12.2%, respectively at 14 days and 14.4% versus 16.1%, respectively at 28 days) (supplementary eTable 3). Remdesivir plus CCS was associated with a significantly lower mortality risk versus CCS alone (*P* < 0.0001) in the early Omicron period, with an aHR (95% CI) at 14 and 28 days of 0.76 (0.72-0.80) and 0.79 (0.76-0.83), respectively, in patients with any supplemental oxygen (Figure 2b).

For patients hospitalized for COVID-19 in the later Omicron period in the PS-matched cohort with any supplemental oxygen (N = 9,950), the unadjusted mortality rate was lower for those administered remdesivir in the first 2 days of hospitalization versus those who did not receive remdesivir during hospitalization (6.6% versus 8.0%, respectively at 14 days and 8.0% versus 9.6%, respectively at 28 days) (supplementary eTable 3). Remdesivir plus CCS was associated with a significantly lower mortality risk versus CCS alone (*P* < 0.0001) in the later Omicron period, with an aHR (95% CI) at 14 and 28 days of 0.75 (0.67-0.83) and 0.76 (0.69-0.83), respectively, in patients with any supplemental oxygen (Figure 2c).

Similar results were obtained with the IPTW sensitivity analysis across all Omicron periods in the study population with any supplemental oxygen (supplementary eTable 6).

## Discussion

The SARS-CoV-2 virus remained one of the top 15 causes of death in the United States in 2024.^20^ Vulnerable patient populations are at continued risk for developing severe SARS-CoV-2 infection. In the current COVID-19 era, these patients are still vulnerable to hospitalization, and while randomized clinical trials are not feasible, real-world evidence can inform treatment decisions made by inpatient providers.

In this study, administration of remdesivir plus CCS versus CCS alone was associated with significantly decreased all-cause in-hospital mortality at 14 and 28 days in patients hospitalized for SARS-CoV-2 infection in the overall, early, and later Omicron periods. This study, including the most recent data available from the later Omicron period, builds on the previous finding that remdesivir plus dexamethasone significantly decreased all-cause in-hospital mortality at 14 and 28 days when compared to dexamethasone alone in patients hospitalized for SARS-CoV-2 infection.^17^

In the PS-matched population in this study, regardless of treatment, unadjusted mortality rates were higher in the early Omicron versus the more recent later Omicron period. Despite the evidence from this study and additional published studies summarized in a systematic review,^6^ 28.2% of the population in the overall Omicron period received CCS alone upon hospitalization for SARS-CoV-2 infection and almost 40% of the group with NSOc were administered CCS alone. The latter strategy is particularly discouraged in light of the RECOVERY trial data that suggest harm with CCS use, rather than the intended benefit, in patients with COVID-19 not yet requiring supplemental oxygen.^16^ Treatment of patients hospitalized for SARS-CoV-2 infection with CCS alone is in apparent contradiction to current evidence that remdesivir treatment confers a significant improvement in all-cause mortality risk.

While we showed a slight decrease in the percentage of patients hospitalized for SARS-CoV-2 infection in the early versus later Omicron period who received CCS alone (30.5% versus 24.1%, respectively), this is still a substantial percentage of patients receiving CCS alone during their hospitalization in the later Omicron period. This presents an opportunity for inpatient providers involved in COVID-19 treatment decisions to monitor and optimize treatment based on the most recent evidence in pursuit of improved clinical outcomes. In particular, hospital-based clinical pharmacists are uniquely positioned to monitor the use of CCS alone, which has been shown to increase mortality in patients with SARS-CoV-2 infection who are not receiving supplemental oxygen.^16^ They also play a critical role in identifying and addressing gaps in treatment guidelines and interpreting real-world evidence to fill those gaps.

Our study has several strengths, including the large population from a multihospital administrative database used in the analysis. In addition, PS matching and IPTW are two well-established methods used in comparative effectiveness research.^19^ However, there are also some limitations of the study. Despite the minimization of residual confounding by matching patients according to PS, age group, admission month, and hospital, there may be residual confounding due to imbalances in unmeasured variables. For example, data on antiviral use or other treatments administered prior to hospitalization, vaccination status of the patients, and onset of symptoms were not available in the database. However, the PS matching approach used to balance measured variables in this study is likely to have at least partially balanced out unmeasured variables such as vaccination and prior infection. All patients included in this study had already progressed to require hospitalization for COVID-19, reflecting impaired protection from any prior immunity, reducing any bias introduced by inclusion of vaccinated versus unvaccinated patients in the analyses. Additionally, subgroup analyses may become increasingly underpowered or fragile, particularly for outcomes that have a smaller absolute risk, such as the risk of mortality in populations with NSOc. Further, reliance on billing and administrative data may introduce misclassification bias, particularly for oxygen use. In addition, since information on treatments initiated prior to the first day of the hospitalization is not available in this database, it is possible that patients in the CCS alone group included those for whom corticosteroid therapy was initiated in an outpatient setting for a different reason and then continued in the inpatient setting irrespective of whether or not it was intended for COVID-19 treatment.

SARS-CoV-2 continues to cause severe disease in vulnerable patient populations and thus remains a public health concern. Therefore, providing and communicating continually updated information on effective treatment options for these patients using real-world evidence remains a priority. This analysis, including the early and later Omicron periods, shows that progress to adopt effective treatments based on evidence-based guidelines has been slow, given that 28.2% of patients hospitalized for SARS-CoV-2 infection did not receive remdesivir in the overall Omicron period. In the early and later Omicron periods, 30.5% and 24.0% of patients hospitalized for SARS-CoV-2 infection, respectively, received CCS alone. While there was a slight decrease in the later Omicron period, still a quarter of patients received CCS alone. Although COVID-19 inpatient management has improved since the early Omicron period, opportunities still exist to optimize care.

## Conclusions

This analysis, including 3 years of data from the Omicron period and a comparison of the early and later Omicron periods, provides additional evidence that remdesivir plus CCS is associated with a signficantly lower mortality compared to CCS alone in patients hospitalized for SARS-CoV-2 infection. This further supports similar findings from previous studies demonstrating the effectiveness of remdesivir in vulnerable patient populations, regardless of their supplemental oxygen requirements, and illustrates the continuing utility of analysis and dissemination of the most recent real-world evidence to help inform treatment recommendations for inpatient providers treating patients with SARS-CoV-2 infection.

## Supplementary Material

zxag034_Supplementary_Data

## Data Availability

The data supporting this study’s findings are available from Premier, Inc. (https://www.premierinc.com/). Restrictions apply to the availability of these data, which were used under license for this study.

## Additional information

The authors provided the following information regarding their contributions to this work: All authors contributed substantially to the manuscript and agreed to the final submitted version.
