# Supplementary material for: Lower mortality risk associated with remdesivir plus corticosteroids vs corticosteroids alone for the treatment of patients hospitalized with SARS-CoV-2 infection in the early and later Omicron periods
Source: Am J Health Syst Pharm. 2026 Feb 9;83(Suppl 3):S2940–51. doi: 10.1093/ajhp/zxag034 (PMC13070680; doi:10.1093/ajhp/zxag034)
Supplement: zxag034_Supplementary_Data [file zxag034_supplementary_data.docx]

## eTable 1. Definitions of key study variables

| Key Study Variables | | Definitions |
| --- | --- | --- |
| Remdesivir treatment | | Billing charges for treatment: Remdesivir;  ICD-10 procedure codes: XW033E5, XW043E5 |
| Corticosteroids treatment | | Billing charges for treatment at baseline: prednisone, prednisolone, methylprednisolone, hydrocortisone, dexamethasone |
| Key Comorbidities | Obesity | ICD-10-CM diagnosis codes: E66, Z6825-Z6845 |
|  | Chronic pulmonary disease | ICD-10-CM diagnosis codes: I278, I279, J40, J41, J42, J43, J44, J45, J46, J47, J60, J61, J62, J63, J64, J65, J66, J67, J684, J701, J703 |
|  | Cardiovascular disease (including hypertension) | ICD-10-CM diagnosis codes: I00-I99 |
|  | Diabetes Mellitus | ICD-10-CM diagnosis codes: E10-E14 |
|  | Renal disease | ICD-10-CM diagnosis codes: I120, I131, N032, N033, N034, N035, N036, N037, N052, N053, N054, N055, N056, N057, N18, N19, N250, Z490, Z491, Z492, Z940, Z992 |
|  | Cancer | ICD-10-CM diagnosis codes: C00-C96 |
| Immunocompromising conditions | | ICD-10-CM code for cancer (C00-C96), transplant (Z94.x), hematologic malignancies (C81.x, C82.x, C83.x, C84.x, C85.x, C88.x, C90.x, C91.x, C92.x, C93.x, C94.x, C95.x, C96.x), primary immunodeficiencies (D80.x, D81.x, D82.x, D83.x, D84.x, G11.3, E70.330, D71.x, D70.x), asplenia (Q89.01, Z90.81), toxic effects of antineoplastics (T45.1x), bone marrow failure/aplastic anemia (D61.x), severe combined immunodeficiencies (D80.x, D81.x, D82.x, D83.x, D84.x, D86.x, D89.0, D89.1, D89.2, D89.3, D89.4x, D89.81, D89.82, D89.89, D89.9), HIV (B20), patients with chronic graft-versus-host disease or who are taking immunosuppressive medications for another indication (Z89.8x, Z79.52, Z79.61, Z79.62x, Z79.63x, Z79.64, Z79.69, Z79.810, Z79.811, Z79.818) |
| Supplemental oxygen requirements | IMV | Billing charges for devices: invasive mechanical ventilation, tracheostomy, endotracheal tube intubation, |
|  | ECMO | Billing charges for devices: extracorporeal membrane oxygenation |
|  | HFO/NIV | Billing charges for devices: negative-pressure ventilation, positive-pressure ventilation, CPAP, BiPAP, high flow system via nasal cannula, venturi face mask, rebreather, non-rebreather mask, positive expiratory pressure |
|  | LFO | Billing charges for devices/oxygen supply: simple face mask, oxygen pendant, low-flow system via nasal cannula, oxygen supply |
|  | NSOc | No billing charges for IMV, ECMO, HFO/NIV, or LFO at baseline |
| Admitting diagnosis | Sepsis | ICD-10-CM diagnosis codes: A02.1, A32.7, A40.x, A41.x, A42.7, A54.86, B37.7, R65.20, R65.21, T81.44X |
|  | Pneumonia | ICD-10-CM diagnosis codes: J12.x, J13, J14, J15.x, J16.x, J17, J18.x, A48.1, B25.0, A37.01, A37.11, A37.81, A37.91, A22.1, B44.0, B77.81, J10.00, J10.01, J10.08, J11.00, J11.08 |
| Baseline medications | Anticoagulants | Billing charges for treatment at baseline: apixaban, argatroban, desirudin, lepirudin, dabigatran, danaparoid, edoxaban, tinzaparin, heparin (excluding use of heparin flush), ardeparin, bivalirudin |
|  | Corticosteroids other than dexamethasone | Billing charges for treatment at baseline: prednisone, prednisolone, methylprednisolone, hydrocortisone |
|  | Convalescent plasma | Billing charges for treatment at baseline: convalescent plasma; ICD-10 procedure codes: XW13325, XW14325 |
|  | Tocilizumab | Billing charges for treatment at baseline: tocilizumab; ICD-10 procedure codes: XW033H5, XW043H5 |
|  | Baricitinib | Billing charges for treatment at baseline: Baricitinib; ICD-10 procedure codes: XW0DXM6, XW0H7M6, XW0G7M6 |
|  | Oral antivirals | Billing charges for treatment at baseline: nirmatrelvir-ritonavir, molnupiravir |
| Abbreviations: BiPAP, bilevel positive airway pressure; CPAP, continuous positive airway pressure; HFO/NIV, high-flow oxygen/non-invasive ventilation; ICD-10-CM, International Classification of Diseases, 10th Revision (Clinical Modification); LFO, low-flow oxygen; IMV, invasive mechanical ventilation; ECMO, extracorporeal membrane oxygenation; NSOc, no supplemental oxygen charges. | | |

## eTable 2. Unadjusted All-cause Inpatient Mortality Rates in the Overall Omicron Period (Crude Population prior to PS Matching)

|  | **Overall Omicron** | | **NSOc** | | **Any Supplemental Oxygen** | |
| --- | --- | --- | --- | --- | --- | --- |
|  | **CCS alone** | **Remdesivir + CCS** | **CCS alone** | **Remdesivir + CCS** | **CCS alone** | **Remdesivir + CCS** |
| **Overall population** | **n=66016** | **n=104900** | **n=26048** | **n=39669** | **n=39968** | **n=65231** |
| 14-day crude mortality rate | 5926 (9.0) | 7430 (7.1) | 1403 (5.4) | 1765 (4.4) | 4523 (11.3) | 5665 (8.7) |
| 28-day crude mortality rate | 7584 (11.5) | 9851 (9.4) | 1741 (6.7) | 2267 (5.7) | 5843 (14.6) | 7584 (11.6) |
| Data is presented as n (%), unless otherwise indicated.  Abbreviations: CCS, corticosteroids; NSOc, no supplemental oxygen charges. | | | | | | |

## eTable 3. Unadjusted All-cause Inpatient Mortality Rates (PS-matching without replacement)

|  | **Early Omicron (Dec 2021 - Dec 2022)** | | **Later Omicron (Jan 2023 - Dec 2024)** | | **Overall (Dec 2021 - Dec 2024)** | |
| --- | --- | --- | --- | --- | --- | --- |
|  | **CCS alone** | **Remdesivir + CCS** | **CCS alone** | **Remdesivir + CCS** | **CCS alone** | **Remdesivir + CCS** |
| **Total study population** | **N = 40712** | **N = 40712** | **N = 17053** | **N = 17053** | **N = 57765** | **N = 57765** |
| 14-day mortality rate | 3984 (9.8) | 3425 (8.4) | 1107 (6.5) | 951 (5.6) | 5091 (8.8) | 4376 (7.6) |
| 28-day mortality rate | 5210 (12.8) | 4709 (11.6) | 1311 (7.7) | 1141 (6.7) | 6521 (11.3) | 5850 (10.1) |
| **NSOc** | **N = 15846** | **N = 15846** | **N = 7103** | **N = 7103** | **n=22949** | **N = 22949** |
| 14-day mortality rate | 950 (6.0) | 848 (5.4) | 307 (4.3) | 292 (4.1) | 1257 (5.5) | 1140 (5.0) |
| 28-day mortality rate | 1204 (7.6) | 1131 (7.1) | 356 (5.0) | 344 (4.8) | 1560 (6.8) | 1475 (6.4) |
| **Any supplemental oxygen** | **N = 24866** | **N = 24866** | **N = 9950** | **N = 9950** | **N = 34816** | **N = 34816** |
| 14-day mortality rate | 3034 (12.2) | 2577 (10.4) | 800 (8.0) | 659 (6.6) | 3834 (11.0) | 3236 (9.3) |
| 28-day mortality rate | 4006 (16.1) | 3578 (14.4) | 955 (9.6) | 797 (8.0) | 4961 (14.2) | 4375 (12.6) |
| Data is presented as n (%), unless otherwise indicated.  Abbreviations: CCS, corticosteroids; NSOc, no supplemental oxygen charges; PS, propensity score. | | | | | | |

## eTable 4. IPTW sensitivity analyses: Total study population

|  | **HR [95% CI]** | ***P*-value** |
| --- | --- | --- |
| **14-day mortality** |  |  |
| Overall Omicron (Dec 2021-Dec 2024) | 0.76 [0.74 - 0.79] | <0.0001 |
| Early Omicron (Dec 2021-Dec 2022) | 0.76 [0.73 - 0.79] | <0.0001 |
| Later Omicron (Jan 2023-Dec 2024) | 0.79 [0.73 - 0.84] | <0.0001 |
| **28-day mortality** |  |  |
| Overall Omicron (Dec 2021-Dec 2024) | 0.79 [0.76 - 0.81] | <0.0001 |
| Early Omicron (Dec 2021-Dec 2022) | 0.79 [0.76 - 0.81] | <0.0001 |
| Later Omicron (Jan 2023-Dec 2024) | 0.80 [0.75 - 0.85] | <0.0001 |
| Abbreviations: CI, confidence interval; HR, hazard ratio; IPTW, inverse probability of treatment weighing. | | |

## eTable 5. IPTW sensitivity analyses: NSOc

|  | **HR [95% CI]** | ***P*-value** |
| --- | --- | --- |
| **14-day mortality** |  |  |
| Overall Omicron (Dec 2021-Dec 2024) | 0.78 [0.73 - 0.84] | <0.0001 |
| Early Omicron (Dec 2021-Dec 2022) | 0.76 [0.70 - 0.82] | <0.0001 |
| Later Omicron (Jan 2023-Dec 2024) | 0.83 [0.73 - 0.95] | 0.0075 |
| **28-day mortality** |  |  |
| Overall Omicron (Dec 2021-Dec 2024) | 0.80 [0.75 - 0.85] | <0.0001 |
| Early Omicron (Dec 2021-Dec 2022) | 0.79 [0.73 - 0.85] | <0.0001 |
| Later Omicron (Jan 2023-Dec 2024) | 0.84 [0.75 - 0.96] | 0.0071 |
| Abbreviations: CI, confidence interval; HR, hazard ratio; IPTW, inverse probability of treatment weighing; NSOc, no supplemental oxygen charges. | | |

## eTable 6. IPTW sensitivity analyses: Any supplemental oxygen

|  | **HR [95% CI]** | ***P*-value** |
| --- | --- | --- |
| **14-day mortality** |  |  |
| Overall Omicron (Dec 2021-Dec 2024) | 0.76 [0.73 - 0.79] | <0.0001 |
| Early Omicron (Dec 2021-Dec 2022) | 0.75 [0.72 - 0.79] | <0.0001 |
| Later Omicron (Jan 2023-Dec 2024) | 0.78 [0.72 - 0.85] | <0.0001 |
| **28-day mortality** |  |  |
| Overall Omicron (Dec 2021-Dec 2024) | 0.79 [0.76 - 0.81] | <0.0001 |
| Early Omicron (Dec 2021-Dec 2022) | 0.78 [0.75 - 0.81] | <0.0001 |
| Later Omicron (Jan 2023-Dec 2024) | 0.80 [0.74 - 0.86] | <0.0001 |
| Abbreviations: CI, confidence interval; HR, hazard ratio; IPTW, inverse probability of treatment weighing. | | |
